# Dermal Matrices: Do We Always Know What Is Going On?

**DOI:** 10.7759/cureus.31979

**Published:** 2022-11-28

**Authors:** Mariana Agostinho, Tatiana Gomes, Vera Eiró, João Nunes da Costa

**Affiliations:** 1 Plastic and Reconstructive Surgery, Centro Hospitalar Lisboa Ocidental, Lisbon, PRT

**Keywords:** contaminated wounds, vesicocutaneous fistula, bioprosthesis, surgimend®, bladder exstrophy

## Abstract

The use of dermal matrices in abdominal wall reconstruction has gained increased attention over time, particularly in contaminated fields. One of their advantages is the greater capacity to resist infection. We report a case of a 36-year-old man, with congenital bladder exstrophy and neobladder reconstruction during childhood. He presented to us with an abdominal hernia associated with a vesicocutaneous fistula. We used a bovine-derived dermal matrix (SurgiMend®, TEI Biosciences, MA, USA) for reinforcement of the abdominal repair considering its laboratory-proven mechanical superiority regarding strength. The early postoperative period was complicated by an infection that led to mesh disintegration and the need for surgical revision. We believe that matrix digestion by bacterial enzymes culminated in rapid breakdown of the product. Further investigations are warranted to determine optimal selection criteria and indications of bioprosthesis in contaminated wounds. Surgeons should be cautious when selecting a biologic mesh in these cases, favoring meshes with a better integration profile.

## Introduction

Reinforcement of a hernia repair with prosthetic materials has significantly reduced rates of recurrence when compared to suture-only techniques. Particularly, the use of dermal matrices in abdominal wall reconstruction has gained increased attention in recent years, since they are theoretically a viable alternative to synthetic meshes in contaminated fields. Incorporation of bioprostheses into host tissue through vascularization is thought to be a key point to resist infection. Moreover, bioprostheses are soft and pliable, which potentially decreases the risk of discomfort and erosion into the bowel. The use of human-derived acellular dermal matrices (H-ADMs) has been discouraged due to unacceptable long-term elasticity [1-3]. Under European legislation (European Community [EC] Directive 2004/23/EC), H-ADMs are otherwise not widely available. This resulted in the introduction of xenografts as biological materials. Nowadays, the most used materials are derived from porcine and bovine tissue. However, few data are currently available to assess long-term durability of bioprosthetic meshes, and their role in abdominal wall reconstruction after bladder exstrophy repair is largely unknown.

## Case presentation

**Figure 1:**
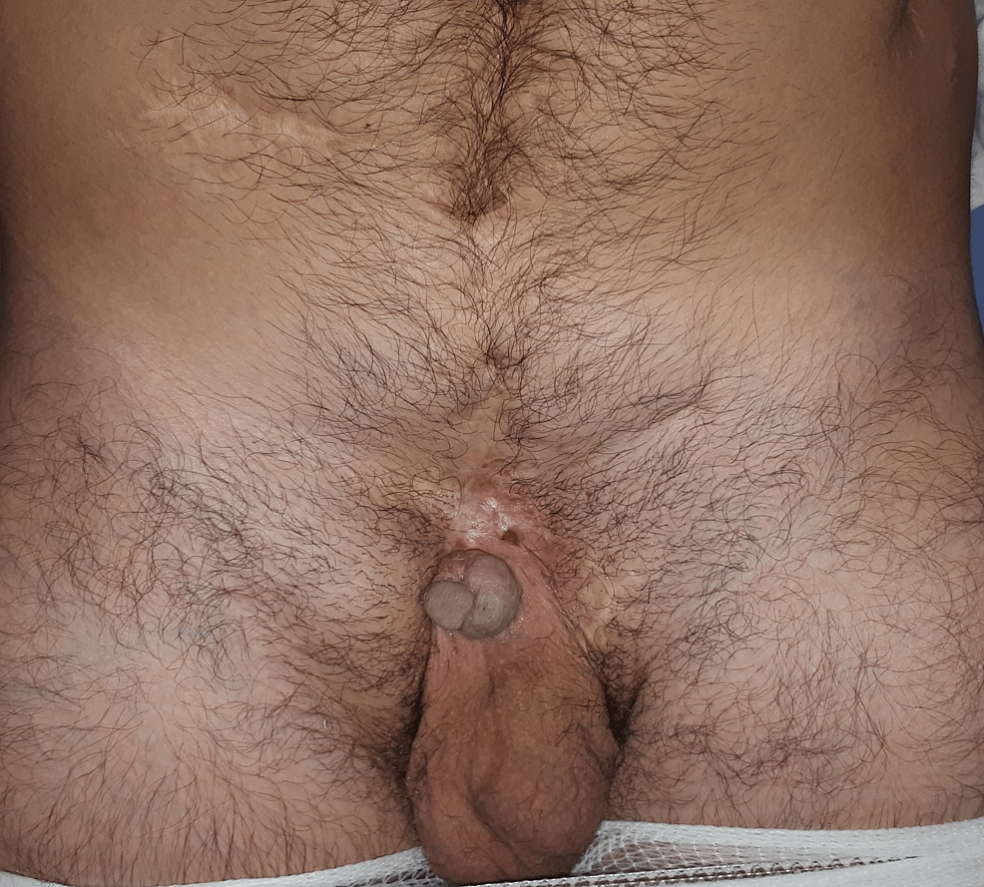
Chronic wound of the abdominal wall Abdominal wall with multiple previous scars and a chronic wound just above the base of the penis

We report a case of a 36-year-old man with congenital bladder exstrophy. During his childhood, in a foreign country, he was submitted to a pelvic osteotomy and neobladder reconstruction with an intestinal segment and urinary diversion into the anal sphincter. The patient also presented a micropenis, hypertension, and renal insufficiency related to recurrent urinary infections. Nineteen years ago, he had a suprapubic herniation of the neobladder that was surgically corrected. At our observation, he complained of a chronic wound of the abdominal wall which had been present for two years. It was associated with a fistulous track that extended from the neobladder to the area above the base of the penis (Figure 1).

**Figure 2:**
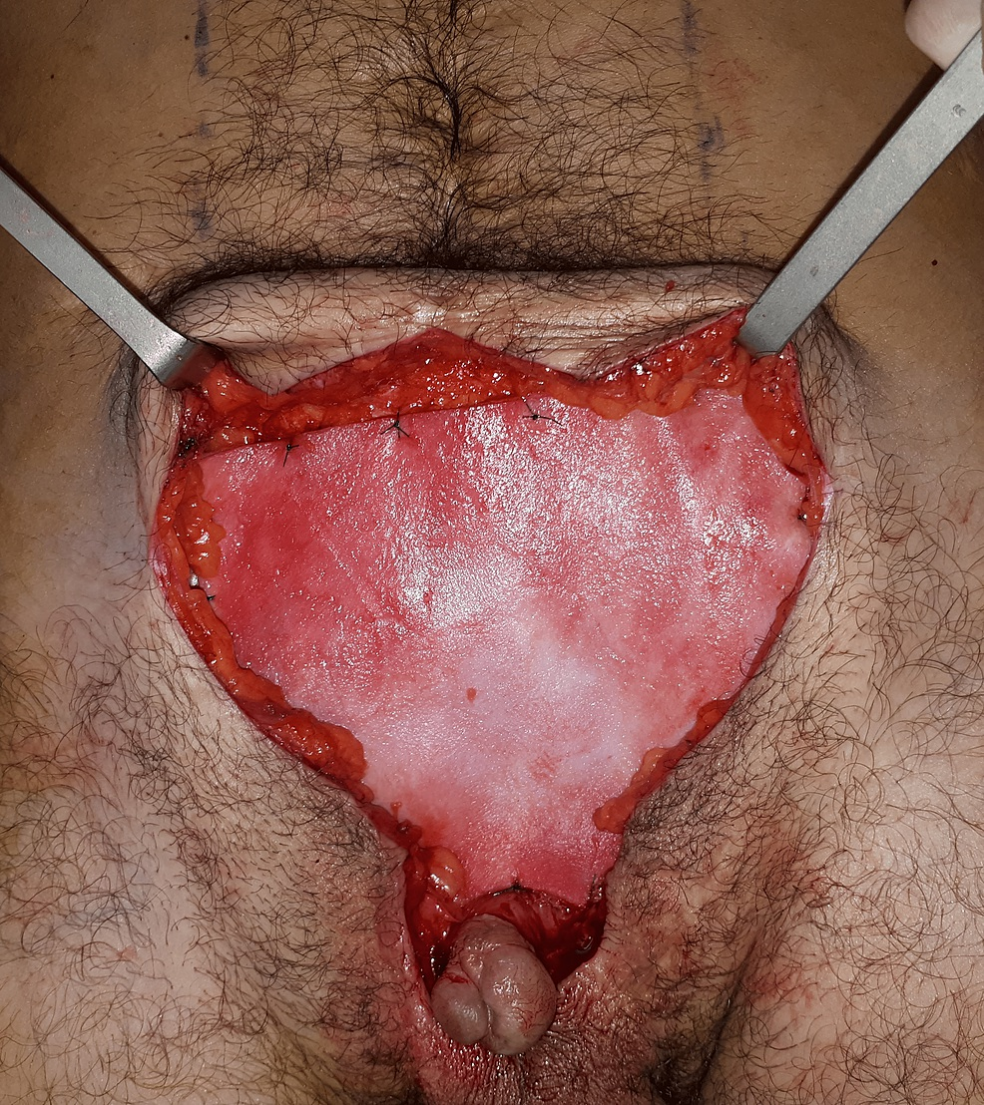
Intraoperative photograph after placing the dermal matrix

We excised the fistulous tract and performed a direct fascial repair and abdominal wall reinforcement with a bovine-derived acellular dermal matrix (Surgimend® 3.0 mm, TEI Biosciences, MA, USA). The matrix was tailored onlay and placed in the infraumbilical region above the penis and laterally fixed to the external oblique muscle and lacunar ligament under no tension (Figure 2).

**Figure 3:**
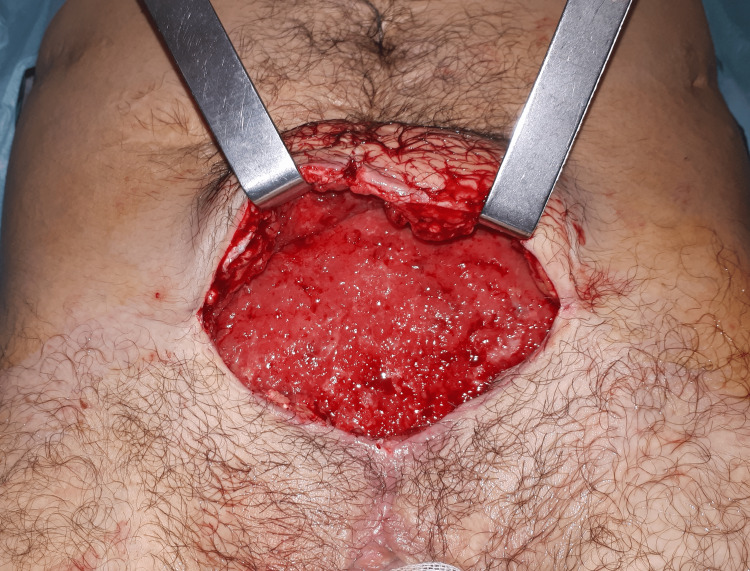
Intraoperative image of mesh disintegration Mesh degradation at the 12th postoperative day.

The surgery was uneventful, and the patient was discharged on the second postoperative day, after drain removal. His early recovery period was complicated by a seroma that infected and led to a re-intervention on the 12th postoperative day. At observation, we noticed that the matrix was coming out piecemeal from the surgical wound. Intraoperatively the mesh appeared degraded and looked like granulation tissue (Figure 3). The surgical cavity was irrigated and *Staphylococcus aureus* and *Trueperella bernardiae* were isolated from the exudate. The patient was prescribed with piperacillin-tazobactam IV therapy and daily wound cleaning with povidone-iodine for 10 days. The drains were then removed, and he was discharged with amoxicillin/clavulanic acid for another 10 days. At three months of follow-up, he was submitted to another surgery to treat a refractory seroma with the application of a fibrin sealant at the surgical wound. At 16 months after the last procedure, the patient no longer had signs or symptoms of hernia or vesicocutaneous fistula recurrence (Figure 3).

## Discussion

There is a wide array of options for abdominal wall reconstruction with biologic meshes. In the current era, porcine and bovine are the most frequently selected. Besides their distinct source, they also differ in their processing methods (decellularization, sterilization, and preservation), which is expected to influence their properties *in situ*.

SurgiMend is a non-cross-linked collagen (type I and III fibers) matrix derived from fetal and neonatal bovine dermis. Its porous architecture enables rapid cell repopulation with host cells and vascularization followed by gradual extracellular matrix remodeling. This is accomplished while maintaining the structural properties of high tensile strength and lack of stretch over time [1]. Laboratory studies have shown that bovine dermal matrices have a higher ultimate tensile strength, suture retention strength, and tear resistance when compared with porcine acellular dermal matrices of equivalent thickness [4]. This grants SurgiMend a theoretical advantage for the reinforcement of the abdominal wall in hernia repair. However, in *in vivo* settings, it is uncertain whether these mechanical properties last. As suggested by Adelman et al., factors related to the surgeon, patient, and material are all potential causes of device failure [4].

In clinical practice, bovine acellular dermal matrices have not yet been extensively studied in abdominal wall reconstruction. To our knowledge, there are only three publications about SurgiMend use in this scenario. Wietfeldt et al. published a small series of five patients in which a bovine acellular dermal matrix was used for abdominal wall reconstruction [5]. They reported three wound-related complications in two patients, both of whom were morbidly obese women with multiple comorbidities including diabetes and malignancy and could not guarantee the maintenance of integrity of the mesh in an infected field. Janfaza et al. in their retrospective review, comparing short-term surgical outcomes of abdominal hernia repair with SurgiMend and a human-derived mesh (FlexHD®, Ethicon, Somerville, NJ, USA), reported a similar number of deep wound infections in the two groups with a lower hernia recurrence rate at one year of follow-up for the SurgiMend group [6]. Clemens et al. directly compared clinical outcomes of the porcine (Strattice™; LifeCell Corporation, Branchburg, NJ, USA) and bovine acellular dermal matrix (SurgiMend) use for non-bridged abdominal wall reconstruction in 120 patients [1]. They concluded that the surgical complication rate in a short term was statistically equivalent although intraoperative device failures were higher in the porcine matrix cohort.

Although theoretically bioprostheses can be salvaged in the presence of infection, the mesh is at risk of being degraded by proteolytic enzymes, decreasing its tensile strength [7]. This can result in device disintegration, which has been already reported in this context with various xenografts [8]. We believe that it was our patient’s case. *Trueperella bernardiae* is a Gram-positive commensal bacillus, found in human skin and the oropharynx. The true incidence and clinical implications of *T. bernardiae* infections are still unknown [9]. On the other hand, it is widely known that nearly all strains of Staphylococcus aureus secrete collagenase [10]. At the second intervention, the mesh was found disintegrated. Currently, the patient is asymptomatic, but a long-term follow-up is needed to adequately assess the efficacy of this biologic mesh’s remnant. The authors think that the reinforcement of the abdominal repair is now only assured by the remaining fibrous tissue. The literature is sparse, but it may be a question of bovine species type or processing conditions that influenced the susceptibility of Surgimend to enzymatic degradation [11].

The importance of biologic meshes in ventral abdominal wall surgery has long been recognized, but significant doubts about their indications in contaminated fields still exist [12]. Now there is still an incomplete understanding of mesh remodeling and changes in mechanical strength over time. Also, the available data are insufficient to direct a patient-specific and cost-effective choice. Repairing contaminated abdominal wall defects is associated with higher morbidity because of the risk of infectious complications that could limit good results. There is a paucity of literature on the use of biologic implants in the exstrophy setting. To the best of the authors’ knowledge, this is the first report of the use of a bovine acellular dermal matrix in bladder exstrophy reconstruction [13-15].

## Conclusions

Biological dermal matrices added a significant improvement to abdominal wall reconstruction and reinforcement. However, further investigations are warranted to determine optimal selection criteria and indications of bioprostheses in contaminated wounds. Even meshes with superior mechanical features may lose their advantageous characteristics in a contaminated field due to enzymatic bacterial action. Surgeons should be cautious when selecting a biologic mesh in these cases, favoring meshes with a better integration profile.
